# Amniotic Membrane Biopolymer for Regenerative Medicine

**DOI:** 10.3390/polym15051213

**Published:** 2023-02-28

**Authors:** Evgeny Milyudin, Larisa Teodorovna Volova, Ksenia E. Kuchuk, Elena V. Timchenko, Pavel E. Timchenko

**Affiliations:** Biotechnology Center "Biotech", Samara State Medical University, 443079 Samara, Russia

**Keywords:** biopolymers, human amniotic membrane, glycerin impregnation, freeze-drying, scanning electron microscopy, Raman spectroscopy

## Abstract

Biopolymers based on the amniotic membrane compare favorably with synthetic materials in that, along with a specific 2D structure, they have biologically active properties. However, in recent years, there has been a tendency to perform decellularization of the biomaterial during the preparation of the scaffold. In this study, we studied the microstructure of 157 samples and identified individual biological components in the manufacture of a medical biopolymer from an amniotic membrane using various methods. Group 1 had 55 samples, and the amniotic membrane was impregnated with glycerol and dried over silica gel. Group 2 had 48 samples, and the decellularized amniotic membrane was impregnated with glycerol followed by lyophilization, Group 3 had 44 samples, and the decellularized amniotic membrane without pre-impregnation with glycerol was subjected to lyophilization. Decellularization was performed by treatment with a low-frequency ultrasound at a frequency of 24–40 kHz in an ultrasonic bath. A morphological study using a light microscope and a scanning electron microscope showed the preservation of the structure of the biomaterial and more complete decellularization in samples subjected to lyophilization without prior impregnation with glycerol. The study of the Raman spectroscopy lines of a biopolymer made from a lyophilized amniotic membrane without preliminary impregnation with glycerin showed significant differences in the intensity of the spectral lines of amides, glycogen, and proline. Additionally, in these samples, the spectral lines of Raman scattering the characteristic of glycerol were not visualized; therefore, only biological substances characteristic of the native amniotic membrane have been preserved.

## 1. Introduction

In regenerative medicine, one of the most unique biopolymers is widely used. It is an amniotic membrane formed by a monolayer of epithelial cells on a basal membrane and a stroma consisting of three layers. Biologically active substances contained in all layers of the amniotic membrane ensure the activation of regenerative processes, cell proliferation, and accelerate cell migration [1,2,3,4]. Currently, amniotic membrane biopolymers are used in the surgical treatment of the consequences of injuries, burns [5,6,7,8,9], in performing reconstructive operations in ophthalmology [10], and as a mechanical substrate for cultured cells [11,12,13].

The composition and structure of the scaffold for fibroblasts used as a cellular component should provide mechanical support and ensure cell viability and growth. Additionally, there should be a number of general properties that determine the possibility of its use, namely, biocompatibility, hypoimmunogenicity, lack of cytotoxicity, and the presence of an adhesive surface for cells. Biological activity is one of the key properties that a scaffold should have. The amniotic membrane fully meets the requirements for the extracellular matrix. Namely, the native amniotic membrane is a polypeptide chain of collagen, laminin, fibronectin, and elastin intertwined with polysaccharide chains of glycosaminoglycan. Laminin, being the main glycoprotein of basement membranes and a thin extracellular matrix, promotes adhesion, growth, migration, and proliferation of cells. Thanks to these properties, the cultured cells are quite simply fixed on the amniotic membrane and continue to develop.

However, studies on the effectiveness of the treated or native amniotic membrane do not stop [3,4]. We propose to use a preserved amniotic membrane as a scaffold. In the process of conservation, cells are destroyed both on the surface and in the structure of the amniotic membrane. In this regard, for greater preservation of the structure of the tissue and cell membranes, treatment with glycerin is performed. However, some researchers note the ambiguous effect of glycerol on cultured cells, namely, a decrease in mitotic activity and an increase in the specific proliferative activity of cells when using biomaterial preserved with glycerol as a matrix [5].

The clinic uses both native and specially processed biomaterial [6,7,8]. Preference is given to amniotic membrane biopolymers. Various methods of biomaterial conservation are used. The most widespread methods of cryopreservation are completed with cryoprotectants, lyophilization, and drying over silica gel [9,10]. During cryopreservation, the viability of the cellular structures of the amniotic membrane is preserved, but the complexity of storage and transportation limits its application [11]. Another common direction in the conservation of biogenic material is the sublimation drying of the amniotic membrane. Along with the convenience of storage, transportation, and use, this process leads to a complete loss of cell viability in the structure of the biomaterial. Previous studies on drying the amniotic membrane over silica gel indicate the preservation of its anatomical and histological structure [11,12]. The preservation of the bionic structure of the material is considered by some authors to be one of the most important factors that ensure the biological activity of this material, while other researchers consider the preservation of viable cells to be optional [12,13,14,15].

Therefore, the question of the preservation of the morphological structure, viable cells, and biologically active substances in a specially treated amniotic membrane remains debatable.

Based on this, we set the following goal in this study.

To study, using a complex of morphological and optical methods, histomorphological features and the preservation of biologically active substances in the amniotic membrane preserved by various methods.

## 2. Materials and Methods

The donor placenta was delivered to the tissue bank 4–6 h after collection. The amniotic membrane was mechanically separated from the chorion and washed from blood clots in physiological saline (0.9% NaCl solution, pH 5–7.5). Fragments of tissue with dimensions of 50 × 50 mm were formed. Further, the donor biomaterial was divided into three experimental groups and one control group:

1. group 1, 55 samples: the amniotic membrane was impregnated with glycerol and placed on frames over silica gel and dried;
2. group 2, 48 samples: the amniotic membrane was treated with a low-frequency ultrasound at a frequency of 24–40 kHz in the Sapphire ultrasonic bath TTK (Sapphire LLC, Moscow, Russia) and impregnated with glycerol followed by lyophilization;
3. group 3, 44 samples: the amniotic membrane was treated with a low-frequency ultrasound at a frequency of 24–40 kHz in the Sapphire ultrasonic bath TTK (Sapphire LLC, Moscow, Russia) and subjected to lyophilization without prior impregnation with glycerol.

The control group consisted of 10 samples of the native amniotic membrane, which were examined without additional processing and drying.

Decellularization of the amniotic membrane was carried out by a physical method, exposure of the biomaterial to low-frequency ultrasound at a frequency of 24–40 kHz in the ultrasonic bath “Sapphire” TTK (LLC “Sapphire”, Moscow, Russia).

The material was lyophilized (vacuum freeze-drying) using an ALPHA2-4LSC freeze-dryer (Martin Christ Gefriertrocknungsanlagen GmbH, Osterode am Harz, Germany). The advantage of this drying method is the absence of high temperatures on the biomaterial, which allows maintaining structural integrity. For additional protection of the biomaterial structure from denaturation, it is possible to use chemicals such as glycerin.

The moisture content of the product was determined using a thermogravimetric infrared moisture meter (Sartorius-MA-150, Malente, Germany).

Morphological studies were carried out after fixing the biomaterial in a 12% neutral formalin solution, passing it through an alcohol battery, and pouring it into celloidin. At least 500 sections were made from different biomaterial samples. Sections were stained with hematoxylin-eosin or picrofuchsin, according to Van Gieson. Image analysis of stained preparations was performed using an imaging system based on an Olympus BX41 research microscope (Olympus, Tokyo, Japan), a ProgRes CF color digital camera, and a desktop computer with Morphology 5.2 software (VideoTesT, Russia).

Scanning electron microscopy (SEM) of the amniotic membrane after preservation was performed using a JEOLJSM-6390 A Analysis Station scanning electron microscope (Japan). For this study, fragments of the biomaterial were washed and fixed with a 2.5% aqueous solution of glutaraldehyde, then the wiring was carried out through a battery of alcohols. After passing through a solution of ethanol in increasing concentration and drying at room temperature for 24 h, gold or carbon was sputtered onto the biomaterial to improve the required surface electrical conductivity when performing scanning electron microscopy.

Raman spectroscopy of preserved amniotic membrane samples was performed at the Department of Laser and Biotechnical Systems of the Samara National Research University. The spectral characteristics of preserved human amniotic membrane samples were studied using an experimental setup including a Shamrock SR-303I high-resolution digital spectrometer (Oxford Instruments PLC, Abingdon, UK) with a built-in Andor DV420A-OE cooled chamber (Oxford Instruments PLC, Abingdon, UK) and a fiber optic probe RPB785 in combination with laser module Luxx Master LML 785.0RB-04 (Laser Components Germany GmbH, Olching, Germany). The spectrograph provided image resolution with a wavelength of 0.15 nm at a low level of intrinsic noise. The laser radiation power of 400 mW within the exposure times used (30 s) did not cause changes in the samples. Raman spectra were recorded using an optical probe, which was located above the object at a distance of 7 mm. In this work, the Raman spectra were analyzed in the range of 380 to 1780 cm^−1^. To eliminate the contribution of autofluorescence in the spectrum, we used the method of subtracting the fluorescent component of the polynomial approximation with additional filtering of random noise effects. The Raman spectra were processed and analyzed using the Wolfram Mathematica 12.2 software package.

The results obtained were processed by the statistical methods of the SPSS_Statistics package. The work was performed with the permission of the Bioethics Committee of the Samara State Medical University (Extract from the protocol No. 206 dated 18 March 2020).

## 3. Results

The feature of the technology for pre-treatment of the donor allogeneic tissues in the Tissue Bank of the Research Institute of BioTech of the Samara State Medical University is the predominant use of physical factors. In particular, low-frequency ultrasonic action makes it possible to remove blood elements, as well as wave action and cavitation ensure decellularization of the amniotic membrane. The lyophilization process and subsequent sterilization by the radiation method make it possible to guarantee the complete absence of viable cellular structures.

The prepared biomaterial retained elasticity by washing using an ultrasonic bath and lyophilization without fixing it on paper. Macroscopically, the biomaterial of all three experimental groups looked like a sheet of tissue paper, velvety to the touch. It should be noted that the samples of group 3 of the lyophilized amniotic membrane without impregnation with glycerol turned out to have a more matte and heterogeneous surface than the samples of groups 1 and 2, pre-impregnated with glycerol.

**Figure 1:**
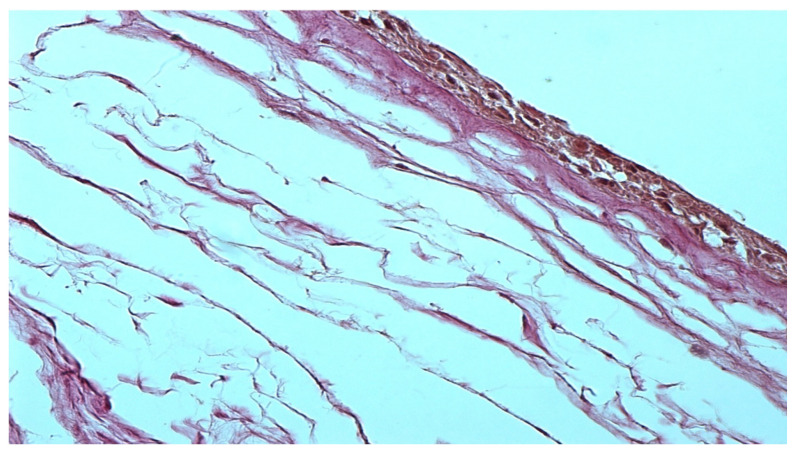
Native amniotic membrane preparation. Stained with picrofuchsin. −×400.

The morphological preparation of the native amniotic membrane presented in Figure 1 demonstrates a completely intact epithelial layer with viable cells and many pinocytic vesicles (indicated by an arrow in Figure 1). A compact layer consisting of densely intertwined collagen fibers is adjacent to the basal membrane, and the fibroblast layer is loose with fibroblasts located in the interweaving of reticular fibers. The spongy layer is represented by delicate, randomly intertwined reticular fibers.

After drying, the biomaterial undergoes significant changes, largely depending on the type of pre-treatment and drying method. In all cases, there is a lack of viable cells both in the epithelial layer and in the stromal layers. The biomaterial dried over silica gel undergoes the greatest structural changes after preliminary impregnation with glycerol.

**Figure 2:**
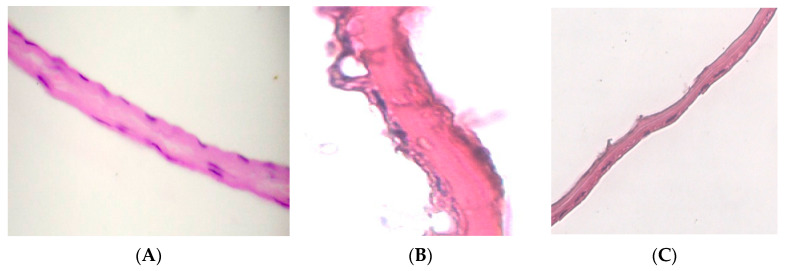
Histological preparations of the amniotic membrane. Staining with hematoxylin-eosin. UV−×400–. (**A**) Amniotic membrane preparation with glycerin before drying over silica gel; (**B**) Amniotic membrane preparation with glycerin before preserved by lyophilization; (**C**) Amniotic membrane preparation without glycerin before preserved by lyophilization.

On histological preparations of the 1st group, a silicified amniotic membrane, after preliminary impregnation with glycerol (Figure 2A), an almost homogeneous band is observed, in which it is difficult to determine the epithelial and stromal layers. In places, strongly flattened cell nuclei are visualized. The compact stroma layer looks like a homogeneous cell-free oxyphilic band. The nuclei of the preserved fibroblasts are rod-shaped. In most cases, shadows of the nuclei are visible in the fibroblast layer. The spongy layer is sharply flattened, determined by differently directed oxyphilic-colored fibers. When morphometry of the total thickness of the preparation of the amniotic membrane dried over silica gel with preliminary impregnation with glycerol, the average measurement value (*n* − 55)–6.9184 μm was obtained.

In a detailed study of histological preparations of group 2, (Figure 2B), the epithelial layer is clearly visualized. In isolated areas, focal destruction of epithelial cells is observed (marked with an arrow). In the preserved cells, pycnosis of the nuclei and the concentration of chromatin in the form of conglomerates is observed. The compact layer, in some places, looks like a homogeneous cell-free oxyphilic band; in some places, fibers are visualized in it. The spongy layer is also preserved but compact, and the structural organization is lost. When we completed the morphometry of the total thickness of preparations of the lyophilized amniotic membrane with pre-impregnation with glycerol, the average measurement value (*n* − 48)–10.236 microns was obtained.

The epithelial layer on histological preparations of the 3rd group of samples (Figure 2B) looks like a flattened homogeneous oxyphilic layer. In single preserved epithelial cells, karyolysis is observed, and the concentration of chromatin is in the form of conglomerates.

The compact layer has a densified homogeneous structure. In the fibroblast layer, we observe a sharp decrease in the number of cellular elements. The nuclei of the preserved fibroblasts are rod-shaped, but in most cases only the shadows of the nuclei are visible. The spongy layer is flattened. When morphometry of the total thickness of the drug lyophilized amniotic membrane without impregnation with glycerol was completed, the average measurement value (*n* − 44)–10.026 microns was obtained.

For a deeper study of the structure of the biopolymer, the state of the surface after exposure to low-frequency ultrasound and drying by various methods, scanning electron microscopy of amniotic membrane samples was performed. Sampling for scanning electron microscopy was carried out from different parts of the placenta. The variability of the picture is also due to different methods of pre-treatment and conservation method.

Taking into account the results of light microscopy, which showed the preservation of the structure of the amniotic membrane after pre-treatment and drying by various methods, we studied the surface of the biomaterial from both sides, both from the epithelial and from the side adjacent to the chorion.

**Figure 3:**
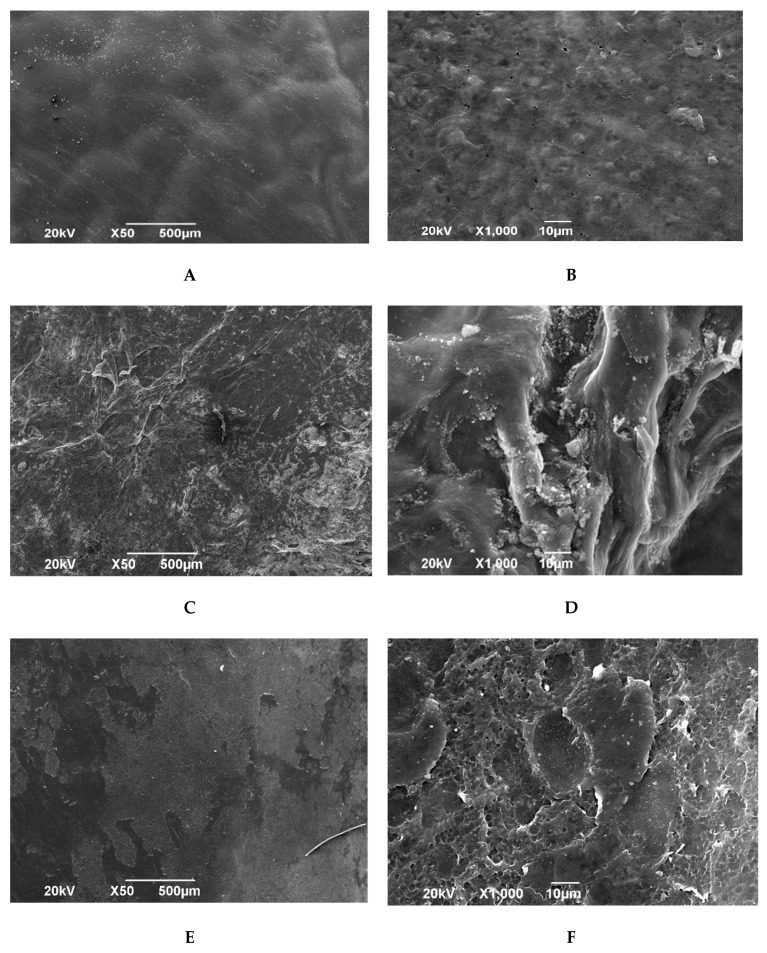
Electron microscopic image of the amniotic membrane in a scanning electron microscope. (**A**) Epithelial surface of an amniotic membrane psreparation pre-impregnated with glycerol and silica-dried. SW. −×50. (**B**) Epithelial surface of a preparation of an amniotic membrane pre-impregnated with glycerol and dried on silica. SW. −×1000. (**C**) Epithelial surface of an amniotic membrane preparation lyophilized with pre-impregnation with glycerol. SW. −×50. (**D**) Epithelial surface of an amniotic membrane preparation lyophilized with pre-impregnation with glycerol. SW. −×1000. (**E**) Epithelial surface of an amniotic membrane preparation lyophilized without glyc erol impregnation. SW. −×50. (**F**) Epithelial surface of an amniotic membrane preparation lyophilized without glycerol impregnation. SW. −×1000.

Scanning electron microscopy of the amniotic membrane dried over silica gel after pre-impregnation with glycerol confirmed the results of the study with a light microscope, namely, the preservation of the epithelial layer (Figure 3A,B). The epithelial layer is represented by convex rounded cells of heterogeneous size. Between individual cells, flat cells of irregular shape are observed. In some areas, cytoplasmic strands are observed. Clearly defined intercellular channels are unevenly distributed over the entire surface. The epithelial layer is adjacent to the basement membrane on the entire surface of the biomaterial.

**Figure 4:**
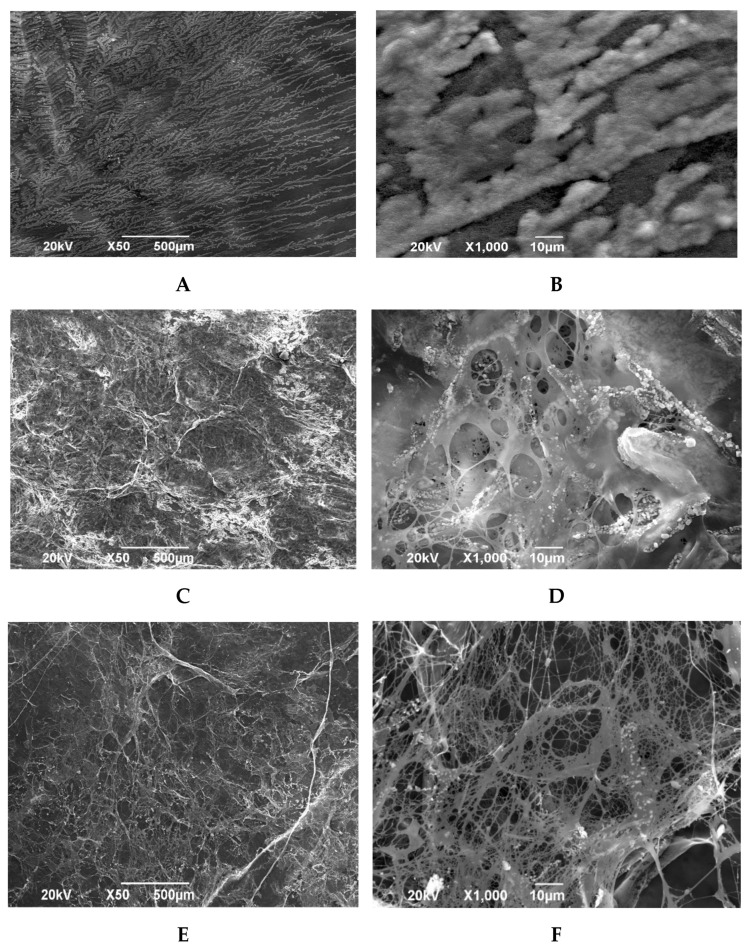
Electron microscopic image of the amniotic membrane in a scanning electron microscope. (**A**) Spongy layer of the preparation of the amniotic membrane, silico-dried and pre-impregnated with glycerol. SW. −×50; (**B**) Spongy layer of the preparation of the amniotic membrane, silico-dried with pre-impregnation with glycerol. SW. −×1000; (**C**) Spongy layer of an amniotic membrane preparation lyophilized with pre-impregnation with glycerol. SW. −×50; (**D**) Spongy layer of an amniotic membrane preparation lyophilized with pre-impregnation with glycerol. SW. −×1000; (**E**) Spongy layer of an amniotic membrane preparation lyophilized without prior soaking in glycerol. SW. −×50; (**F**) Spongy layer of an amniotic membrane preparation lyophilized without prior soaking in glycerol. SW. −×1000.

The spongy layer of the amniotic membrane lyophilized with preliminary impregnation with glycerin preserves the structure of loose reticular tissue. Multidirectional connective tissue fibers give a volumetric structure to the biomaterial. Some connective tissue fibers are connected to each other by homogeneous structureless substrates attached to them in clusters (Figure 4C,D).

The spongy layer of amniotic membrane samples lyophilized without prior impregnation with glycerol looks the least damaged. The loose structure of the reticular connective tissue is preserved, and some connective tissue fibers are connected to each other, on which single structureless substrates are fixed. On these preparations, the through-porosity of the biomaterial is clearly visible. It should be noted the presence of only single homogeneous formations attached to collagen fibers (Figure 4E,F).

**Figure 5:**
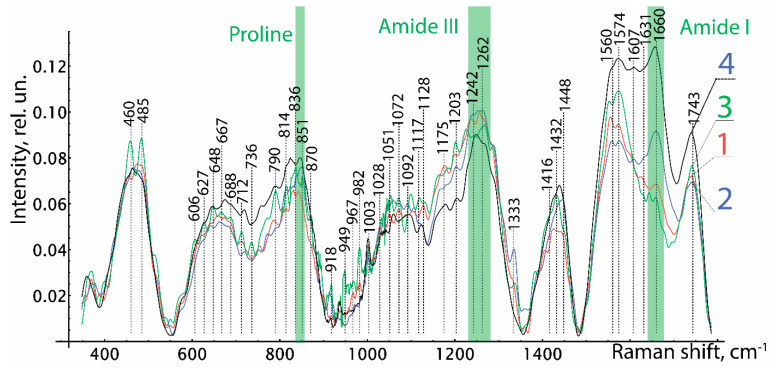
Averaged Raman spectra of the studied samples of the amniotic membrane. 1-the first group of the studied samples, 2-the second group of the studied samples, 3-the third group of the studied samples, and 4-native samples.

Analysis of the Raman spectra in the range of 380 to 1780 cm^−1^ confirmed the safety of biologically active substances in the preserved amniotic membrane, as can be seen in Figure 5. The Raman spectra of the studied groups of biomaterial samples have spectral differences in the entire range. In contrast to the spectra of groups 1 and 2, in group 3 we can clearly see the Raman scattering lines of 485 cm^−1^ (Glycogen), 648 cm^−1^ (C-Ctwistingmodeoftyrosine), 836 cm^−1^ Glucose-saccharide-band (overlapswithacyl band), 949 cm^−1^ (single bond stretching vibrations for the amino acids proline and valine and polysaccharides), 982 cm^−1^ (CH bending (lipids)), 1432 cm^−1^ (CH2 deformation), and 1560–1580 cm^−1^ (Amide II (Parallel/Antiparallel β-sheet structure).

Relative line intensities differ greatly 1660 cm^−1^ (Amide I vibration (collagen-like proteins)) between groups 2 and 1, 3. Differences in other Raman lines are not so important. When comparing the Raman spectra of the native amniotic membrane with the spectra of the differently preserved amniotic membrane, a significant difference was obtained between the biomaterials of groups 1 and 2 and similar in manifestation to the spectra of the native biomaterial and the amniotic membrane lyophilized without glycerin treatment. It should be noted that experiments carried out on the epithelial and stromal sides of the amniotic membrane did not reveal differences in the Raman spectra, and this is explained by the insignificant thickness and the transparent porosity of the biomaterial formed as a result of processing. As a result of the Raman spectroscopy, we found out the safety of collagen, peptides, and amides of the preserved amniotic membrane in all samples of the amniotic membrane. However, it is necessary to note the pronounced spectrum 606–630 cm^−1^ characteristic of glycerol in samples of 1 and 2 groups of biomaterial. Additionally, in group 3, in comparison with groups 1 and 2, more intense lines of the Raman spectrum 485, 648, 790, 836, 949, 982, 1432, 1560–1580 cm^−1^ are observed.

## 4. Discussion

The conducted comprehensive study showed that scanning electron microscopy allows a much more complete study of the state of the surface of the biopolymer and, therefore, assumes the preservation of biologically active properties. In particular, both in the study of preparations using a light microscope and in scanning electron microscopy, it was determined that the special treatment of the amniotic membrane without the use of an ultrasonic bath but necessarily impregnated with glycerol before silica drying ensures the preservation of the cellular elements of the epithelial layer, the basement membrane, and at the same time contributes to greater compaction of the stromal layers.

Exposure of the amniotic membrane to low-frequency ultrasound on biomaterial samples in groups 2 and 3 leads to complete destruction of cellular structures and partial removal of cellular components. There is also significant damage to the epithelial layer with desquamation of the basement membrane, exposure of the stroma, and the formation of trabecular architectonics. These changes are more pronounced in samples of the amniotic membrane, which is exposed to low-frequency ultrasound without impregnation with glycerol before lyophilization. In these samples, the epithelial layer and most of the basement membrane is completely removed.

In the study of biological tissue samples subjected to lyophilization with preliminary impregnation with glycerol, significant damage to cellular structures and destruction of the epithelial layer and basement membrane are also observed. In this group of amniotic membrane samples, less pronounced changes in the stromal layers are observed. The layers are less compacted, the reticular fibers are loosely arranged, and the number of homogeneous structureless substrates along the multidirectional fibers is significantly less than on the preparations of the 1st group of the amniotic membrane subjected to drying over silica gel.

The stromal layers of the amniotic membrane not impregnated with glycerol before lyophilization are the least susceptible to compaction, the reticular fibers are multidirectional, and the porous structure of the biomaterial is traced. Single homogeneous structureless substrates are observed along the multidirectional reticular fibers.

Physical methods of influencing biological tissue are expected to have an impact on cell viability, which is confirmed by our study. The epithelial layer is destroyed and desquamated. However, biologically active substances are preserved in the samples we study, which we have confirmed in a non-destructive way, i.e., Raman spectroscopy. Therefore, it can be assumed that biologically active substances can be preserved not only in cellular structures, but also in the stroma and intercellular substance.

For the use of the amniotic membrane as a substrate for cell cultivation and the creation of tissue engineering complexes, a decellularized biomaterial is more preferable. Therefore, a lyophilized amniotic membrane without pre-impregnation with glycerol is more preferable, since with this method of preparing the biopolymer, the epithelial layer is more completely removed. When considering this biopolymer as a scaffold, the question remains on which surface it is more efficient to fix cultured cells.

We have confirmed the content and preservation of biologically active substances in a biopolymer made from a specially processed amniotic membrane. The use of freeze-drying allows preserving the original properties of an allogeneic biomaterial without disturbing the structure of proteins. Our comparative study using Raman spectroscopy of native and three groups of amniotic membrane samples after different methods of pretreatment and preservation by different methods showed some spectral differences in all groups.

A comparative analysis of the allogeneic biomaterial untreated with glycerin revealed changes in the spectra on the correlation scattering lines 485 cm^−1^, 648 cm^−1^, 790 cm^−1^, 836 cm^−1^, 949 cm^−1^, 982 cm^−1^, 1432 cm^−1^, and 1560–1580 cm^−1^ compared to biopolymers treated with glycerol. The predominance of the intensity of the spectra of 485 cm^−1^ and 648 cm^−1^ in the study of the lyophilized amniotic membrane without treatment with glycerol, even in comparison with the native amniotic membrane, suggests a higher content of glycogen, proline, and tyrosine in the biopolymer structure and the formation of phosphodiester bonds between them. At the same time, the retention of the organic matrix in all samples should be noted, as evidenced by the presence of Raman scattering lines at 850 cm^–1^ (Proline), 1238 cm^–1^–1272 cm^–1^ (Amide III), 1450 cm^–1^, and 1600 cm^−1^ (Amid I). A significant predominance of secondary amides in the native and lyophilized amniotic membrane without treatment with glycerol, represented by cyclic peptides, suggests a greater preservation of the protein structures of the biopolymer. These results allow us to assert that the biological activity of biological preparations is preserved, which is more pronounced in samples without preliminary impregnation with glycerol, despite the decellularization performed by physical methods.

The preservation of biologically active substances makes it possible to consider a biopolymer based on a lyophilized allogeneic amniotic membrane without preliminary impregnation with glycerol, the most effective for use as a biocoating and scaffold in regenerative medicine.

## 5. Patents

This section is not mandatory but may be added if there are patents resulting from the work reported in this manuscript.

## Data Availability

Not applicable.
